# Belly dancer syndrome improved by withdrawal of droxidopa and amantadine

**DOI:** 10.1002/ccr3.7237

**Published:** 2023-04-22

**Authors:** Mihiro Kaga, Hiroyoshi Ueda, Chika Sunagawa, Masashi Oe, Atsushi Shima, Nobuyasu Matsukawa, Takeshi Ueda

**Affiliations:** ^1^ Emergency and General Internal Medicine Rakuwakai Marutamachi Hospital Kyoto Japan

**Keywords:** abdominal wall, amantadine, dopamine, droxidopa, dyskinesia, movement disorders

## Abstract

A man hospitalized for cerebral infarction developed drug‐induced belly dancer syndrome, which improved after withdrawal of droxidopa and amantadine. Drugs that modulate dopamine neurotransmission have been reported to be associated with this syndrome. When belly dancer syndrome is suspected, clinicians should consider drug‐induced abdominal dyskinesia and medication withdrawal.

The patient was a man in his 80s hospitalized for cerebral infarction. He had cerebrovascular parkinsonism treated with 200 mg of droxidopa and 100 mg of amantadine. Involuntary abdominal contractions (Video S1) were observed during hospitalization, which indicated belly dancer syndrome.^1^ Droxidopa and amantadine administration were terminated; 3 days after, involuntary abdominal movements completely disappeared (Video S2). Drugs that modulate dopamine neurotransmission have been reported to be associated with it.^2^, ^3^ In the present case, amantadine may have been associated with these involuntary abdominal movements. Clinicians should consider drug‐induced abdominal dyskinesia and medication withdrawal.

## AUTHOR CONTRIBUTIONS

**Mihiro Kaga:** Conceptualization; data curation; investigation; resources; software; writing – original draft; writing – review and editing. **Hiroyoshi Ueda:** Data curation; resources; supervision. **Chika Sunagawa:** Data curation; resources; supervision. **Masashi Oe:** Data curation; investigation; resources; supervision. **Atsushi Shima:** Data curation; resources. **Nobuyasu Matsukawa:** Conceptualization; data curation; investigation; resources; supervision. **Takeshi Ueda:** Supervision.

## FUNDING INFORMATION

None.

## CONFLICT OF INTEREST STATEMENT

The authors have no conflicts of interest to declare.

## CONSENT

Written informed consent was obtained from the patient to publish this report in accordance with the journal's patient consent policy.

## Supporting information


Video S1

Video S2

## Data Availability

The data that support the findings of this study are available from the corresponding author upon reasonable request.
